# Crimean–Congo Hemorrhagic Fever Virus in Africa: Epidemiological Trends, Transmission Ecology, Hotspot Heterogeneity, and Preparedness Challenges—A Narrative Review

**DOI:** 10.3390/tropicalmed11060161

**Published:** 2026-06-16

**Authors:** Elichilia Robert Shao, Jeremia J. Pyuza, Tito Kibona, Laura Shirima, Eliaichi A. Mlay, Alice Andongolile, Ray Kayaga, Semvua Kilonzo, Blandina T. Mmbaga, Jaffu Chilongola

**Affiliations:** 1 Internal Medicine Department, Kilimanjaro Christian Medical Centre, P.O. Box 3010, Moshi 25119, Tanzania; 2 Kilimanjaro Clinical Research Institute, P.O. Box 2236, Moshi 25119, Tanzania; 3 Internal Medicine Department, School of Medicine, KCMC University, P.O. Box 2240, Moshi 25119, Tanzania; 4 Pathology Department, School of Medicine, KCMC University, P.O. Box 2240, Moshi 25119, Tanzania; 5 Department of Life Sciences and Bio-Engineering, Nelson Mandela African Institution of Science and Technology, P.O. Box 447, Arusha 23301, Tanzania; 6 Department of Epidemiology and Biostatistics, School of Public Health, KCMC University, P.O. Box 2240, Moshi 25119, Tanzania; 7 Department of Prosthetics and Orthotics, School of Rehabilitation Medicine, KCMC University, P.O. Box 2240, Moshi 25119, Tanzania; 8 Community Health Department, Kilimanjaro Christian Medical Centre, P.O. Box 3010, Moshi 25119, Tanzania; 9 Tanzania Veterinary Laboratory Agency, P.O. Box 447, Arusha 23301, Tanzania; 10 Internal Medicine Department, Catholic University of Health and Allied Sciences, P.O. Box 1464, Mwanza 33100, Tanzania; 11 Pediatric and Child Health Department, School of Medicine, KCMC University, P.O. Box 2240, Moshi 25119, Tanzania; 12 Department of Medical Biochemistry and Molecular Biology, School of Medicine, KCMC University, P.O. Box 2240, Moshi 25119, Tanzania; jaffu.chilongola@kcmcu.ac.tz

**Keywords:** Crimean–Congo haemorrhagic fever, One Health, hotspots, *Hyalomma*, Africa, surveillance, epidemiological patterns, outbreak preparedness

## Abstract

**Background:** Crimean–Congo hemorrhagic fever virus (CCHFV) is an important tick-borne zoonosis and an emerging public health threat across Africa. Although evidence of viral circulation is mounting, information remains fragmented, limiting a comprehensive understanding of transmission ecology, regional hotspot heterogeneity, and preparedness needs across the continent. **Methods:** This narrative review critically synthesized published literature on CCHFV in Africa, identified through PubMed, Scopus, and Google Scholar and supplemented by citation tracking and authoritative public health reports. Evidence from epidemiological, ecological, molecular, surveillance, and One Health studies was integrated to examine transmission dynamics, geographic hotspot distribution, viral diversity, risk factors, diagnostic and surveillance challenges, and preparedness strategies. **Results:** Available evidence shows marked geographic heterogeneity in CCHFV transmission across Africa, with hotspot regions shaped by ecological suitability, Hyalomma tick distribution, livestock–human interactions, and health system capacity. Livestock consistently show higher exposure than humans, underscoring their role as key indicators of viral circulation. Diagnostic limitations, passive surveillance, ecological variability, and serological cross-reactivity contribute to substantial under recognition of disease burden, while molecular studies reveal considerable viral diversity and ongoing evolution across African regions. **Conclusions:** CCHFV remains underdiagnosed and underreported in many African settings because of limited surveillance and diagnostic capacity. Strengthening integrated One Health surveillance, expanding laboratory and genomic capacity, utilizing livestock as sentinel populations, and improving cross-sectoral collaboration are critical for enhancing early detection, outbreak preparedness, and effective public health response across the continent.

## 1. Introduction

CCHF is a severe viral zoonosis that poses severe public health challenge globally [1]. First identified in Crimea in 1944 and later in the Congo in 1956, the CCHF virus is a member of the genus *Orthonairovirus* within the family Nairoviridae [2]. The virus is primarily transmitted to humans through the bite of infected *Hyalomma* ticks or via direct contact with the blood or tissues of viremic animals or patients [3,4]. Clinical manifestations in humans range from nonspecific febrile symptoms to severe multi-organ failure, with reports indicating a significant case fatality rate of 10% to 40% [1]. While some observations suggest a potential geographic expansion of CCHFV, these trends may partly reflect improved diagnostic detection and enhanced surveillance efforts rather than a purely biological spread [5]. Evidence suggests that the distribution of the virus is closely tied to the ecological suitability of its primary *Hyalomma* vectors [3]. Previous data underscore a disparity in viral circulation in Sub-Saharan Africa where in some settings an average human seroprevalence of 13.6% was found, whereas livestock—which often remain asymptomatic—show a significantly higher average seroprevalence of 44.3% [6]. Kenyan data has suggested the seroprevalence reaches 89.7% in camels and 53.9% in cattle, compared to 7.2% in the local human population [7]. Similarly, preliminary data in Mauritania showed that livestock seroprevalence has been recorded as high as 74.6% in cattle and 89.4% in dromedaries [7]. The CCHFV genome exhibits high genetic diversity, and in Africa, the virus is primarily represented by Lineages I, II, and III, as well as the Africa 4 lineage [6]. Identifying suspected CCHFV cases is challenging because symptoms overlap with common tropical febrile illnesses. Limited diagnostic capacity and healthcare worker awareness hinder diagnosis [8]. Improving training, diagnostics, and surveillance is crucial for early detection and control [9]. The management of CCHF in Africa is hampered by the lack of licensed vaccines and specific antiviral therapies [1,10]. These factors indicate that the true disease burden is likely underestimated due to passive surveillance and the prevalence of subclinical infections [2,10]. The high prevalence of CCHFV in livestock, especially cattle and camels, underscores the need for a One Health approach to disease surveillance. Integrating animal, human, and environmental surveillance enables early detection, targeted public health actions, and improved outbreak preparedness, thereby reducing human infections and limiting future outbreak [11].

## 2. Methods

This narrative review provides a critical synthesis of the literature on Crimean–Congo hemorrhagic fever virus (CCHFV) in Africa, focusing on epidemiological trends, transmission ecology, heterogeneity of hotspots, and preparedness challenges. Relevant literature was identified through searches of major electronic databases, including PubMed, Scopus, and Google Scholar, using keyword combinations such as “Crimean-Congo hemorrhagic fever”, “CCHFV”, “Africa”, “Hyalomma”, “One Health”, “surveillance”, “seroprevalence”, “outbreak”, and “zoonotic transmission”. Studies were selected for their relevance to the review’s objectives and included original research articles, surveillance reports, outbreak investigations, ecological studies, molecular epidemiology reports, and systematic reviews that provided evidence of CCHFV circulation in humans, livestock, ticks, or wildlife in African settings. Additional references were identified using Mendely soft ware for mac through citation tracking of relevant publications and authoritative reports from international public health agencies (https://mendeley.en.softonic.com/mac accessed on 20 May 2026).

Rather than conducting a formal systematic review or meta-analysis, evidence was critically appraised and synthesized narratively to identify recurring epidemiological patterns, regional heterogeneity, transmission pathways, surveillance capacity, diagnostic challenges, and preparedness strategies across Africa. Particular attention was given to studies describing human–animal–tick interfaces, ecological determinants of transmission, One Health implementation, and evidence supporting the identification of CCHFV hotspot regions. The synthesized evidence was organized into thematic domains, including virology and transmission ecology, epidemiological patterns, geographic hotspots, risk factors, surveillance and diagnostic systems, One Health approaches, prevention and control strategies, and research priorities. This approach enabled the integration of diverse evidence sources while highlighting knowledge gaps and opportunities to strengthen CCHFV preparedness and response across the African continent.

## 3. Background and Theoretical Foundations

### 3.1. Viral Genomics and Diversity

CCHF is a negative-sense, single-stranded RNA virus within the *Nairoviridae* family. Its tripartite genome—comprising Large (L), Medium (M), and Small (S) segments—facilitates significant genetic plasticity [12]. Within Africa, phylogeny based on the S segment suggests three major lineages, though global diversity includes at least 4 lineages [4]. Recent investigations in Senegal and Mauritania highlight the virus’s capacity for reassortment; for instance, a 2022 Senegal tick strain exhibited a reassortant profile with L and M segments from genotype I and an S segment from genotype III [13]. This genomic variability poses ongoing challenges for universal diagnostic assays and vaccine candidates [12,13].

### 3.2. Defining Epidemiological Hotspots and Ecology

In this review, “hotspots” are defined as geographical regions where there is a documented convergence of viral circulation (molecular or serological evidence in humans or animals), high density of competent *Hyalomma* vectors, and frequent human–animal interfaces. The primary transmission cycle remains centered on *Hyalomma* ticks, particularly *H. marginatum rufipes* and *H. impeltatum* in West Africa [2]. While livestock—specifically cattle, sheep, and goats—are recognized as possible amplifying hosts, they typically remain asymptomatic [13]. Cattle consistently show high seroprevalence, often ranging from 14% to 80% across various African biomes [2,14]. Emerging data from Kenya indicate that peridomestic rodents may also participate in the transmission cycle, with a reported 6.5% seroprevalence suggesting a role as supplementary hosts [15].

### 3.3. Clinical Impact and Recent African Outbreaks

CCHF presents a severe clinical progression, with case fatality rates typically ranging between 10% and 40% [1,16,17]. Recent evidence suggests that mortality can be significantly higher in specific outbreak contexts; for example, the 2022 outbreak in Podor, Senegal, recorded an unprecedented mortality rate where two out of three confirmed cases resulted in death (66.7%) [18]. Similarly, reports from Uganda indicate mortality rates can reach approximately 30% by the second week of illness. These figures highlight the urgent need for early detection, as the infection often presents initially with non-specific symptoms like high fever and myalgia before progressing to a hemorrhagic phase characterized by multi-organ failure [19].

### 3.4. Diagnostic Realities and the One Health Approach

Reliable diagnosis in Africa remains constrained by limited molecular diagnostic capacity, shortage of trained personnel, inadequate biosafety infrastructure and restricted access to reference laboratories capable of performing confirmatory testings [14]. The recent detection of CCHFV in previously unrecognized areas in Côte d’Ivoire may indicate wide distribution [20]. Addressing these gaps requires a unified One Health approach that integrates human, veterinary, and environmental surveillance to mitigate the impact of emerging zoonotic threats [21,22,23].

### 3.5. One Health Framework

The complex ecology of CCHF, involving *Hyalomma* ticks, livestock, wildlife, and humans, necessitates a One Health approach that integrates human, animal, and environmental health sectors [2]. This framework recognizes that effective prevention requires coordinated surveillance across species, integrated vector management, and multisectoral policy development to address the asymptomatic nature of infection in animals, thereby facilitating “silent” spread [24,25]. While several African countries have begun implementing One Health platforms, significant gaps in coordination and resource allocation persist [26]. Evidence suggests that for these platforms to be effective, they must transition from reactive outbreak response to proactive monitoring of vector distribution and livestock seroprevalence at key domestic-wild animal interfaces [27].

## 4. Epidemiological Patterns in Africa

### 4.1. Historical Context and Temporal Trends

CCHF has been recognized in Africa for over six decades, with 494 cases and 115 deaths reported across the continent between 1956 and 2020 [2]. Historical underestimation likely resulted from limited diagnostic capacity and misdiagnosis as other febrile illnesses as well as cross-reactivity [28]. Improved surveillance and diagnostic capacity may partially explain the apparent increase. For example, nine African countries have reported their first human cases only since 2000 [2]. In Uganda, the detection of multiple sporadic outbreaks since 2013 suggests a persistent viral presence within the “Cattle Corridor” [24]. This pattern highlights that, while the geographic range appears to be expanding, the lack of longitudinal data makes it difficult to distinguish between “emerging” hotspots and “newly recognized” endemic areas [2].

### 4.2. Incidence and Prevalence Data

#### 4.2.1. Human Disease Burden

Precise incidence data for human CCHF in Africa remain limited due to surveillance gaps. In Uganda’s 2018–2019 outbreaks, 14 confirmed cases were identified across 11 districts [23]. In Nigeria, a systematic review analysis indicates a pooled human seroprevalence of 8.7%, though individual studies in northern regions have reported widespread unrecognized exposure [29].

#### 4.2.2. Livestock Seroprevalence and Cross-Reactivity

Livestock seroprevalence offers a broader view of viral circulation. Pooled data from Nigeria indicate a 39.5% prevalence in animals. In Kwara State, 71.9% of cattle were seropositive for CCHFV antibodies. However, interpreting these high rates requires care due to serological cross-reactivity with other nairoviruses, specifically the Dugbe virus [21,28]. Recent investigations show that while immunofluorescence assays (iIFA) frequently produce cross-reactive results, N-protein-based ELISAs are more specific for differentiating CCHFV from DUGV [21,26]. Evidence indicates that 37.9% of cattle in certain Nigerian regions are co-exposed to both viruses, underscoring the need for species-adapted, validated diagnostic tools to avoid overestimating CCHF burden [21,29].

#### 4.2.3. Summary of Regional Hotspots and Preparedness

The following table summarizes the key available evidence and gaps identified across major African contexts (Table 1).

### 4.3. Molecular Epidemiology and Viral Diversity

Phylogenetic analyses identify that African CCHFV strains typically cluster into three major lineages (Africa 1, 2, and 3). In Kenya, viral sequences from sheep and rodents show high nucleotide identity (96–98%) to the Africa 3 lineage [2]. Reassortment is a notable feature of CCHFV evolution, which can lead to the emergence of variants with altered virulence or transmissibility. Ongoing molecular surveillance is critical for monitoring these changes and ensuring that diagnostic primers remain effective against diverse regional genotypes [30].

**Table 1:** Comparative synthesis of major Crimean–Congo hemorrhagic fever hotspot regions, epidemiological evidence, risk factors, and preparedness gaps across Africa.

Region	Country	Evidence Type	Main Hotspot/Zone	Key Epidemiological Findings	Major Preparedness Gaps	Key References
East Africa	Uganda	Human outbreaks, livestock serology, molecular detection	Cattle Corridor	Recurrent outbreaks; ~73% of confirmed cases during 2018–2019 outbreaks occurred in the cattle corridor; CFR ~36%; strong livestock–human interface	Weak district preparedness; delayed diagnosis; limited decentralized response systems	[31,32]
East Africa	Kenya	Livestock serology, molecular detection, rodent surveillance	Isiolo, Maasai Mara, pastoral ecosystems	Camel seroprevalence 89.7%; cattle seroprevalence >50% in some settings; widespread silent circulation across pastoral systems	Under-detection in mobile pastoral systems; fragmented surveillance	[6,33]
East Africa	Tanzania	Human and livestock serology	Northern Tanzania	Human exposure among febrile patients; livestock-associated transmission risk in pastoral ecosystems	Limited integrated surveillance and molecular diagnostics	[34,35]
West Africa	Senegal	Human outbreaks, tick surveillance, molecular detection	Podor, Kedougou	Podor outbreak CFR 66.7%; repeated emergence; molecular confirmation in humans and ticks	Limited surge capacity; delayed decentralized detection	[13,18,36]
West Africa	Mauritania	Integrated One Health surveillance; livestock serology	Southern pastoral regions	Extremely high livestock seroprevalence (cattle 74.6%, dromedaries 89.4%); strong One Health evidence	Weak cross-border coordination and surveillance continuity	[7,11]
West Africa	Nigeria	Human and livestock serology	Northern Nigeria	Pooled livestock seroprevalence 39.5%; cattle seroprevalence up to 71.9%; extensive regional circulation	Diagnostic cross-reactivity; fragmented surveillance systems	[17,22,28,37]
West Africa	The Gambia	Livestock serology	Rural livestock systems	Serological evidence in cattle and small ruminants suggests silent circulation	Sparse surveillance and limited outbreak preparedness data	[38]
Central Africa	Democratic Republic of the Congo	Human and livestock serology	Livestock–farmer interfaces	Serologic evidence among livestock and farmers; likely underrecognized endemic transmission	Weak diagnostic infrastructure and surveillance coverage	[39,40]
	Cameroon	Molecular and serological evidence	Tick–livestock ecosystems	Molecular and serological evidence in livestock and ticks indicates active circulation	Limited molecular surveillance and ecological monitoring	[4,41]
Central Africa	Gabon	Human serology	Rural populations	First detection of CCHFV antibodies in rural populations; possible geographic expansion	Sparse epidemiological data and limited preparedness evidence	[42]
Northeast Africa	Sudan	Human outbreaks, nosocomial transmission	Multiple outbreak regions	Multiple epidemics and documented healthcare-associated outbreaks between 2010–2020	IPC weaknesses and outbreak containment challenges	[43,44]
Southern Africa	South Africa	Human outbreaks, occupational exposure, molecular detection	Livestock industry regions	Longstanding endemic hotspot; occupational transmission among animal-industry workers; molecular detection in ticks	Persistent occupational exposure risk despite stronger infrastructure	[2,45]
Southern Africa	Malawi	Livestock serology	Smallholder livestock systems	Cattle seroprevalence among smallholder farmers suggests active circulation	Limited preparedness and surveillance data	[42]

## 5. Geographic Hotspots and Spatial Distribution

**Figure 1:**
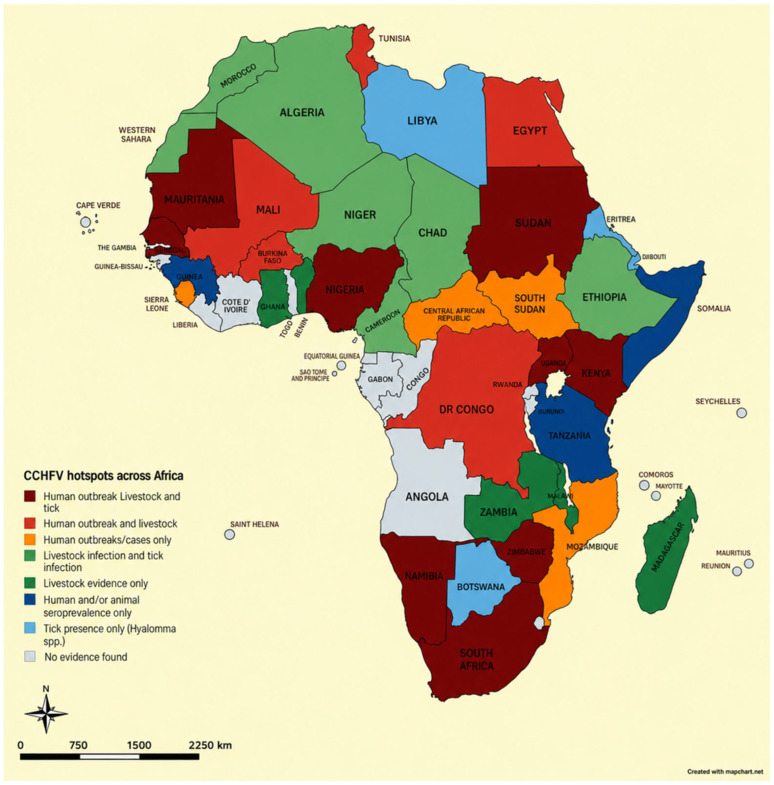
The geographic distribution of identified African CCHFV hotspot regions, along with supporting epidemiological evidence, is shown in this map.

For the purpose of this review, CCHFV hotspots were operationally defined as areas exhibiting one or more indicators of active or potential transmission, including recurrent human outbreaks, documented human cases, elevated human or livestock seroprevalence, molecular detection of CCHFV in humans, livestock or ticks, persistent *Hyalomma* tick presence, livestock infection, healthcare-associated transmission events, geographic clustering of cases, or combined ecological and epidemiological evidence supporting sustained transmission [36,37,46]. Areas with serological evidence alone or vector presence alone were also included as potential hotspots because they indicate silent circulation or ecological suitability for future emergence [2,9,15]. While CCHF appears widely distributed, current data suggest that transmission intensity is heterogeneous across the continent [2,43,44,47]. The geographic distribution of identified African hotspot regions, along with supporting available epidemiological evidence, is summarized in the hotspot map (Figure 1).

### 5.1. East African Hotspots

Uganda and Kenya represent the most documented hotspots in East Africa. Uganda’s “Cattle Corridor” is a critical region for CCHFV, where intensive livestock–human contact drives risk [6,24]. Available evidence from Kenya, Uganda, and northern Tanzania suggests sustained circulation of CCHFV within several East African pastoral ecosystems, although the true burden of human infection remains uncertain because of limited surveillance and underdiagnosis [6,24,31,32,33,34,35]. The relatively small number of reported human cases despite high livestock seroprevalence may reflect low spillover rates, substantial under-detection, or a combination of both factors [24,48]. This suggests CCHFV is endemic in East African pastoral systems, this pattern may reflect low spillover, under-detection, or both [24,32,35].

### 5.2. West African Hotspots

West Africa has emerged as an important hotspot for CCHFV, with increasing evidence from countries such as Senegal, Mauritania, and Nigeria suggesting expanding endemic circulation [11,13,18]. Recent outbreaks in Senegal, particularly in the Podor region, have highlighted the potential for severe disease and demonstrated that multiple independent viral introductions can occur within the same geographic area, underscoring the dynamic and evolving epidemiology of CCHFV [13,18]. This highlights that while the virus is endemic, localized clusters can be highly virulent. Nigeria is a key hub for CCHFV, especially in northern states [29]. Evidence from a systematic review indicates substantial CCHFV circulation among livestock in sub-Saharan Africa, with especially high levels of exposure reported in cattle from Nigeria [37]. This underscores the role of livestock as important reservoirs of viral transmission and highlights the need for integrated One Health surveillance strategies [9,25]. However, caution is needed; data show up to 37.9% of livestock might be co-exposed to Dugbe virus, which often causes cross-reactivity in tests [21,28]. This suggests that “hotspot” status in Nigeria might be partially inflated by other nairoviruses, necessitating more specific diagnostic protocols [29].

### 5.3. Central and Southern African Hotspots

Data from Central Africa are patchy, but findings in the DRC and Cameroon suggest active yet underrecognized transmission [39,40,41]. The detection of antibodies in Gabon extends evidence of exposure into previously understudied areas [42]. In Southern Africa, South Africa is the main hotspot, mainly affecting animal industry workers [2]. Unlike East and West Africa’s pastoralist-driven dynamics, Southern Africa’s transmission is more localized, aided by advanced surveillance that offers better case-to-exposure matching [2,46,47].

### 5.4. Spatial Risk Mapping and Environmental Suitability

Spatial analyses show that CCHF risk is highest in areas with moderate temperatures, sufficient humidity for Hyalomma ticks, and dense livestock populations [10,49]. Mapping highlights the Sahel, East African Cattle Corridor, and Southern Africa’s pastoral regions as most vulnerable [2,17]. However, the evidentiary base for these maps remains uneven due to the diversity of presented data as per methodology [47]. While suitability is high across sub-Saharan Africa, the transition from “suitable environment” to “active hotspot” depends on local human–animal interfaces and the capacity of regional health systems to detect “silent” viral circulation before human spillover occurs [6,9,49,50].

## 6. Risk Factors and Drivers of Transmission

The transmission dynamics of CCHF in Africa are driven by a complex interplay of ecological, behavioral, and systemic factors [51]. However, the evidence base remains geographically uneven, and many current interpretations are based on localized studies that may not be universally applicable across the continent’s diverse biomes [2].

### 6.1. Biological and Ecological Drivers: The Tick–Livestock Interface

The presence of competent *Hyalomma* vectors remains the primary ecological determinant of CCHF distribution [52]. While cattle are often described as the “principal” amplifying hosts, evidence indicates significant variation in seroprevalence that reflects local ecological heterogeneity [53]. Livestock Seroprevalence: Cattle consistently exhibit the highest exposure rates, with studies in Nigeria reporting a pooled seroprevalence of 39.5%, while specific regions such as Kwara State report rates as high as 71.9% [21]. The Cross-Reactivity Challenge: High seroprevalence in livestock must be interpreted with caution. Recent data suggest that standard assays often fail to distinguish CCHFV from Dugbe virus [28]. In Nigerian cattle, up to 37.9% of animals show evidence of co-exposure, suggesting that some “hotspots” may be over-identified due to serological cross-reactivity [21,28]. Wildlife Involvement: Emerging evidence from Kenya suggest that the transmission cycle may have extended beyond domestic ruminants; peridomestic rodents have shown a 6.5% seroprevalence, suggesting they may act as supplementary reservoirs in certain landscapes [2,15].

### 6.2. Human Exposure: Occupational and Socio-Behavioral Interfaces

Human risk is concentrated at specific interfaces where direct contact with infected blood or ticks is frequent. Rather than being a generalized “sporadic threat,” CCHF risk in Africa is highly localized to specific occupational groups [2]. Occupational Risk: Animal husbandry, slaughtering, and veterinary work are the most consistently identified risk factors [12]. In Uganda’s 2018–2019 outbreaks, case–control analyses identified that handling meat from sick animals was significantly associated with infection [23]. Similarly, in South Africa, risk is primarily concentrated among workers in the commercial cattle industry [45]. Behavioral Drivers and Poverty: Socio-cultural practices—such as manual tick removal (often by crushing), eating engorged ticks and slaughtering sick animals without PPE [19,32,33,54,55,56]. Even in the household where economy is much better the traditional of not using protective gears might be stronger than the economic factors. In many African pastoralist communities, the high cost of acaricides results in inadequate tick control, thereby maintaining high viral loads in domestic herds [3,57,58].

### 6.3. Systemic and Environmental Risk Factors

The transition from viral circulation to recognized human outbreaks is often mediated by healthcare capacity and climatic shifts [59]. Nosocomial Transmission: Healthcare settings represent a critical vulnerability [60]. Inadequate infection prevention and control measures [61], and a lack of PPE have led to documented nosocomial clusters in Sudan [43,44]. The non-specific early symptoms of CCHF often lead to diagnostic delays, increasing the risk of hospital-acquired transmission [8]. Environmental Suitability: Spatial mapping suggests that CCHFV risk peaks in areas with moderate temperatures and seasonal rainfall patterns that favor *Hyalomma* survival [10,61]. In African settings, Kenya, transmission intensity is closely linked to seasonal shifts that drive tick activity [2]. However, it remains unclear whether recent “increases” in geographic range reflect true environmental expansion or simply the implementation of better surveillance tools in previously neglected regions [53,62]. Clinical Outcomes and Mortality: While general fatality rates are cited at 10–40%, recent data suggest that localized outbreaks can be more severe [1]. The outbreak in Senegal’s Podor region underscored the devastating consequences of delayed case detection and response, illustrating how CCHFV outbreaks can result in severe clinical outcomes when surveillance and early diagnostic capacity are insufficient [18]. These interconnected risk factors suggest that preparedness must move beyond “aspirational” goals to context-specific interventions, such as improving acaricide access for pastoralists [63].

## 7. Surveillance and Diagnostic Systems

### 7.1. Current Surveillance Infrastructure

Surveillance systems for CCHFV across Africa remain heterogeneous, reflecting disparities in public health infrastructure [2]. Most endemic regions rely on passive healthcare-based reporting of undifferentiated febrile or hemorrhagic illness, inherently limited by poor access, low clinical suspicion, and delayed laboratory confirmation [1,63,64]. Substantial clinical overlap with malaria, dengue, bacterial sepsis, typhoid, and other viral hemorrhagic fevers leads to frequent misclassification and underestimation of true burden [34,63,64,65], particularly in rural pastoral community [35]. Countries with recurrent outbreaks, such as Uganda, have developed relatively functional systems including reporting networks and molecular diagnostics [31,32], although gaps persist in remote districts Senegal’s 2022 outbreaks demonstrated both gains and vulnerabilities in detection [13,18], with high mortality suggesting earlier undetected transmission [53]. Surveillance performance depends on laboratory availability, healthcare worker awareness, reporting efficiency, and outbreak investigation timeliness [65]. Many systems remain project-based and externally funded rather than institutionalized, raising sustainability concerns as activities decline after funding ends [1,25]. Consequently, surveillance across Africa is predominantly reactive, identifying outbreaks only after severe human disease rather than through integrated animal, vector, or community monitoring in the One Health approach model [1,2,24].

### 7.2. Laboratory and Molecular Diagnostic Capacity

Laboratory diagnostic capacity remains a primary bottleneck for effective CCHF surveillance and outbreak response across Africa [66,67]. Molecular confirmation via RT-PCR requires specialized infrastructure, high-level biosafety containment (BSL-3/4), and trained personnel—requirements that are difficult to sustain in the rural and pastoral regions where outbreaks typically occur [1,68]. The centralization of these services in urban reference laboratories necessitates long-distance sample transport, resulting in delays that hinder clinical management, contact tracing, and infection prevention [1,29]. Beyond infrastructure, decentralization is obstructed by reagent shortages, irregular electricity, and inconsistent quality assurance [69]. While serological assays are more accessible and effective for human and livestock seroepidemiology [6,13], they are less reliable for acute diagnosis because antibodies may be undetectable during the early, highly infectious stage [19]. However, integrated diagnostic strategies—combining molecular and serological testing across human, animal, and vector populations—have proven valuable for mapping viral circulation in Senegal [18,36], and Uganda [19]. The lack of localized diagnostic capacity directly limits epidemiological visibility; CCHF is frequently misdiagnosed as malaria or typhoid, leading to underreporting and uneven continental data [69,70,71,72]. Addressing these disparities requires sustained investment in regional infrastructure, biosafety systems, and workforce training [1]. Given competing public health priorities, strengthening capacity must involve phased, context-specific approaches that balance technological feasibility with long-term sustainability [1].

### 7.3. Integrated One Health Surveillance

The complex epidemiology of CCHF requires integrated surveillance of human, animal, vector, and environmental data within a One Health framework [1,2,73]. Despite this necessity, surveillance in many African regions remains fragmented, with health, veterinary, and environmental sectors often operating under separate institutional structures and funding streams [74]. This institutional siloing creates significant operational barriers, particularly a lack of data interoperability between ministries, which prevents early warning signals in livestock or vectors from triggering timely public health responses [24,75].

The impact of these gaps is evident in East African pastoral systems. While livestock seroprevalence reaches up to 89.7% in some Kenyan communities, surveillance remains predominantly focused on human case detection, missing opportunities for early viral identification through animal or tick monitoring [7,15,76]. However, successful models exist: Mauritania and Senegal have utilized multisectoral investigations—combining human case data with livestock and entomological surveys—to achieve a more comprehensive understanding of viral circulation and risk factors [7,11]. While countries like Uganda and Nigeria have made progress in cross-sectoral collaboration and proactive risk assessment, significant challenges persist regarding decentralization and the sustainability of these efforts beyond active outbreak periods [17,22,28]. Operationalizing One Health in resource-constrained settings is often hindered by limited financial resources, a lack of shared digital platforms, and a reliance on external, research-oriented funding rather than institutionalized national systems [1,77]. Strengthening CCHF control will require transitioning from reactive, outbreak-driven coordination to long-term institutional integration capable of sustaining multisector collaboration [1,78].

### 7.4. Livestock and Entomological Surveillance

Livestock and entomological surveillance are critical but underdeveloped components of CCHF monitoring in Africa [9]. Because livestock act as amplifying hosts and *Hyalomma* ticks are the primary vectors, animal surveillance underestimates viral circulation and delays outbreak detection [23,30]. Evidence suggests transmission is far more widespread than animal- case counts indicate and may include wild and domestic animals [79,80].

#### 7.4.1. Livestock Serosurveillance

Livestock serosurveillance identifies areas of possible viral circulation even where human outbreaks are rare [7]. In Uganda, nationwide seroprevalence is estimated at 31.4% [46]. Studies in Kenya report seropositivity as high as 53.9% in cattle and 89.7% in camels [6,19], while northern Tanzanian cattle show rates of 49.6% [16]. These data suggest livestock can serve as early-warning indicators for human infection risk [7]. However, most surveillance remains fragmented, research-driven, and poorly integrated into routine veterinary systems [9,39]. Barriers include resource constraints (funding, personnel, and laboratory support) and the difficulty of monitoring mobile pastoralist herds [81,82,83].

#### 7.4.2. Entomological Surveillance and Modeling

Vector monitoring is largely reactive and typically triggered by human cases [17]. Investigations in Senegal demonstrated the value of integrated assessments using RT-PCR to identify viral circulation in *Hyalomma* tick populations, though these efforts often follow human transmission [14]. To complement field monitoring, spatial risk models that incorporate climate, vegetation, and livestock density identify potential transmission zones [84]. While useful for prioritizing resources, these models require “ground-truthing” via tick collection and virological testing for validation [16].

#### 7.4.3. Tick Control and Resistance

Emerging acaricide resistance—specifically to synthetic pyrethroids and organophosphates—poses a growing threat to CCHFV control across sub-Saharan Africa [84]. Effective prevention requires locally adapted strategies that account for the socioeconomic differences between sedentary and pastoral systems [85,86]. Strengthening CCHF early warning systems necessitates integrating veterinary infrastructure with public health systems and moving toward sustainable, routine surveillance [86,87].

### 7.5. Community-Based and Early-Warning Surveillance Systems

Community-based and syndromic surveillance systems are underutilized but critical early-warning components for CCHF in Africa [24]. In pastoral and rural settings, disease signals often emerge outside formal healthcare; thus, relying solely on facility-based passive surveillance risks delayed recognition due to geographic, economic, and sociocultural barriers [24,88]. Syndromic surveillance of hemorrhagic clusters can improve detection, yet CCHF’s nonspecific symptoms—overlapping with malaria, dengue, and other febrile illnesses—limit specificity and increase the risk of missed cases [24,62,63,64]. As demonstrated in Uganda, laboratory confirmation is essential to guide responses in regions with co-circulating febrile etiologies [1,24,62]. Furthermore, operational success is constrained by inconsistent digital tool usage, workforce shortages, and infrastructure deficits in remote districts [1,89].

Community-based surveillance (CBS) offers a vital complement in areas with frequent livestock and tick exposure [80]. Engaging community health workers and livestock owners leverages local knowledge of animal health and tick patterns to identify unusual illness patterns before they become clinically apparent in formal systems [80,88]. However, structured CBS for CCHF remains rare and often fragmented across Africa [65]. Success requires seamless integration with formal infrastructure, effective risk communication to address stigma or diagnostic misconceptions, and sustained community trust [24]. Digital and mobile-health technologies may strengthen these systems by accelerating communication between remote communities, veterinary services, and health authorities [89]. Nevertheless, their feasibility depends on infrastructure, digital literacy, and long-term financial sustainability [90]. Ultimately, the efficacy of CBS and syndromic surveillance depends on their integration with laboratory diagnostics, functional reporting pathways, and broader health-system strengthening [1,89,90].

### 7.6. Surveillance Gaps and Operational Limitations

Despite CCHF’s threat in Africa, gaps in surveillance and diagnostics hinder accurate burden assessment and timely response [6]. Systems are often fragmented, geographically uneven, and reliant on external funding rather than sustainable infrastructure [6]. Underreporting is a key issue; mild or asymptomatic cases often bypass healthcare, underestimating true burden [2,9]. In remote areas, fatalities are misattributed, obscuring transmission patterns [9]. Reported cases reflect diagnostic access more than true prevalence [2]. Lack of integration between human, veterinary, and entomological sectors weakens early warning, as data sharing is inconsistent [90,91]. Systems are reactive, detecting outbreaks only after severe disease appears [11]. Many programs are donor-driven, risking workforce loss and data gaps when funding ends [6]. Low clinical suspicion and endemic diseases like malaria cause delayed reporting [92]. Solutions include decentralized diagnostics, sustainable funding, and integrated surveillance for proactive outbreak management [1]. Collectively, these surveillance biases and persistent data gaps create major uncertainties in understanding the true epidemiology and geographic burden of CCHF across Africa, as summarized in Table 1.

## 8. Outbreak Preparedness and Response Gaps

### 8.1. National Preparedness and Outbreak Response Systems

CCHF preparedness across Africa is inconsistent and insufficiently institutionalized, often characterized by reactive mobilization rather than sustained, specific preparedness frameworks [1,24]. While several nations have general viral hemorrhagic fever guidelines, few possess operational CCHF-specific plans that integrate laboratory response, clinical management, and veterinary coordination into a unified One Health structure [24]. Uganda maintains one of the continent’s most developed systems, largely due to recurrent exposure to VHFs such as Ebola and CCHF [69]. This experience has bolstered its rapid response, laboratory networks, and epidemiological investigation capacity [36,50]. However, maintaining these systems outside active outbreak periods and decentralizing capacity to peripheral districts remain significant operational hurdles [92]. This highlights a broader trend where preparedness often only improves following repeated exposure, leaving regions with infrequent outbreaks vulnerable to delayed recognition [24,91,92].

Senegal’s 2022 outbreaks further illustrated these vulnerabilities. Despite successful mobilization and livestock surveillance, high mortality rates and simultaneous emergences in geographically distinct regions revealed gaps in early detection and surge capacity that strained national infrastructure [18,36]. In contrast, Nigeria has utilized qualitative risk assessments to identify preparedness gaps proactively within a One Health framework [29]. However, translating these assessments into operational readiness remains difficult in resource-constrained environments where implementation capacity is limited [93]. Effective epidemic containment is often hindered by systemic delays, including weak referral systems, transportation barriers, and late laboratory confirmation [24]. These challenges are exacerbated by porous borders and the transboundary movement of livestock and ticks, which facilitate regional transmission while coordination mechanisms remain inconsistently operationalized [8]. Furthermore, many systems rely heavily on external donor funding and emergency mobilization, compromising the long-term sustainability of workforce capacity and health security infrastructure [1]. Currently, CCHF preparedness remains uneven and operationally fragile, necessitating a shift from episodic response toward integrated, institutionalized preparedness [1,24].

### 8.2. Clinical Management and Healthcare System Readiness

Effective CCHF management in Africa relies on early recognition, rapid supportive care (e.g., coagulopathy correction), and strict infection prevention and control, as no universally proven antiviral therapy exists [93]. Case fatality rates vary significantly across the continent, ranging from 10 to 40% in Ugandan outbreaks to exceptionally high rates in events such as Senegal’s Podor outbreak [18,20,24]. These disparities might reflect differences in healthcare infrastructure and diagnostic timing rather than viral virulence alone [1]. Diagnostic delays are a primary challenge, as patients initially present with nonspecific febrile illness often misdiagnosed as malaria or bacterial infections [76]. System readiness is further constrained in rural and district-level facilities by chronic shortages of trained personnel, laboratory support, blood products, and personal protective equipment [1]. These gaps, compounded by long referral distances and weak transportation systems in pastoral settings, increase both mortality risks and opportunities for community or nosocomial transmission [4]. Furthermore, many healthcare workers have limited familiarity with CCHF due to the sporadic nature of outbreaks, leading to low clinical suspicion and delayed isolation [62,94]. While the efficacy of ribavirin, vaccine remains a subject of ongoing debate due to inconsistent clinical evidence, high-quality supportive care remains the cornerstone of management [93,94]. Strengthening critical care capacity, transfusion services, and fluid management is prioritized over pharmaceutical interventions [95,96]. Ultimately, improving CCHF outcomes in Africa requires sustained investment in emergency care infrastructure, workforce development, and rural healthcare capacity [1,97].

### 8.3. Infection Prevention and Control Systems

Infection prevention and control is a cornerstone of CCHF management due to frequent healthcare-associated transmission in endemic regions [98]. The high viral load in the blood and body fluids of severely ill patients poses a substantial occupational risk to healthcare, laboratory, and mortuary personnel, particularly when cases are unrecognized or IPC measures are inconsistent [99]. For example, a South African nosocomial outbreak resulted in 33% of exposed healthcare workers developing the disease via needle-stick injuries and nearly 9% through direct contact with infected fluids [100]. Effective IPC requires rapid case identification, isolation, appropriate personal protective equipment, safe specimen handling, environmental decontamination with chlorine-based agents, and safe burial practices [100,101,102,103]. However, implementation remains uneven across African healthcare systems due to infrastructure disparities and resource limitations [1,101]. While tertiary hospitals may have better IPC capacity, they often face overcrowding and supply chain disruptions [102]. Conversely, district and rural facilities frequently lack formal training, PPE, running water, and reliable waste management [94,95]. Delayed recognition of CCHFV significantly elevates transmission risks [104,105]. Because early symptoms often mimic malaria or bacterial sepsis, infected patients may be admitted to general wards where invasive procedures and emergency resuscitation occur without isolation precautions [106,107]. Such facilities can act as amplifiers; in one Mauritanian outbreak, an unrecognized index case led to 15 secondary infections among staff and visitors [108]. Sustainability is often cyclical, with preparedness declining between outbreaks. Strengthening IPC systems for CCHF provides broader regional health security benefits but requires long-term investment in infrastructure, workforce training, and supply chain resilience [1].

### 8.4. Tick Control and Environmental Preparedness

Tick control is a critical yet operationally neglected component of CCHF prevention in Africa [109,110]. Because *Hyalomma* ticks are the primary vectors and livestock serve as major amplifying hosts, effective vector management including one health approach is essential to reducing human exposure [88]. However, current programs are often reactive and inconsistently implemented across endemic regions [24,111,112]. In Uganda, outbreaks are frequently linked to high tick exposure in grazing fields [31,32], yet management responsibilities often fall to resource-poor farmers who may lack the training or funds for consistent acaricide usage [19]. Weak veterinary infrastructure and livestock mobility in pastoral systems further complicate sustained monitoring and intervention [113]. A major barrier to effective control is the emergence of acaricide resistance; investigations in Uganda have identified resistance rates of 13% for amitraz and up to 90% for certain synthetic pyrethroids [84,114]. Consequently, sustainable preparedness requires community-level strategies, including coordinated spraying, acaricide rotation, and livestock-owner education [79]. Preparedness must also incorporate ecological risk mapping, as landscape factors such as the prevalence of shrub and grassland significantly influence vector distribution [115]. Climate change is further expanding Hyalomma habitats and altering seasonal activity across Africa, thereby heightening risk at the human–livestock–wildlife interface [116]. Transitioning from reactive interventions to long-term operational investment and integrated veterinary infrastructure is vital for mitigating future spillover [24].

### 8.5. One Health Operational Preparedness and Cross-Border Coordination

The multisectoral ecology of CCHF necessitates an operational One Health framework that integrates human health, veterinary, environmental, and border-control sectors [1,24]. Because CCHF transmission involves complex interfaces between livestock, tick vectors, and wildlife, isolated sector-specific responses are insufficient for effective outbreak management [117]. Despite policy-level recognition, operational implementation in Africa remains inconsistent, constrained by institutional fragmentation and the separation of human and animal health systems into parallel structures with divergent priorities [118]. Consequently, multisectoral collaboration is often reactive—occurring during active outbreaks—rather than sustained through continuous preparedness and integrated surveillance [24]. Retrospective investigations demonstrate the value of integration: Mauritania successfully combined human, livestock, and tick surveillance data to clarify transmission dynamics [7,11], while Senegal’s response to the Podor outbreak utilized simultaneous livestock and entomological investigations to guide interventions [13,18]. Similarly, assessments in Uganda emphasize that formal partnerships between health and agricultural ministries are essential for effective vector control and preparedness in livestock-dependent communities [119].

Many endemic countries lack formal One Health platforms with the requisite authority, funding, or legal frameworks to mandate cross-sectoral data sharing [26].These challenges are exacerbated at international borders, where transboundary livestock trade and pastoral migration facilitate the dissemination of infected ticks, yet response mechanisms remain nationally compartmentalized [8]. Financial sustainability and workforce limitations further undermine preparedness. Many One Health initiatives rely on external donor funding rather than stable domestic financing, leaving them vulnerable when funding cycles end [6,74]. Furthermore, competing priorities (e.g., HIV/AIDS, malaria) and a lack of multidisciplinary training pathways for personnel hinder the development of a workforce capable of conducting integrated outbreak investigations [119]. To improve CCHF preparedness, African nations must transition from ad hoc, donor-dependent coordination to institutionalized multisectoral systems capable of sustained surveillance and response [6,74].

### 8.6. Emerging Risks, Research Gaps, and Future Preparedness Threats

The future landscape of CCHFV in Africa is being reshaped by evolving ecological, epidemiological, and operational factors. While current outbreaks remain geographically heterogeneous, the convergence of environmental change, livestock mobility, and persistent infrastructure gaps suggests an increasing risk of transmission across the continent [8,112].

#### 8.6.1. Ecological and Environmental Drivers

Climate change is a primary driver of emerging risk, as shifts in temperature and humidity alter the geographic distribution and seasonal activity of *Hyalomma* tick vectors [116]. Ecological niche modeling indicates that rising temperatures may facilitate vector establishment in currently low-risk regions while intensifying transmission in endemic areas through extended activity seasons [2,4,41]. Concern arises from migration to high-density peri-urban areas, where rural livestock in crowded cities raises the risk of human exposure [120,121].

#### 8.6.2. Anthropogenic and Biological Factors

Livestock mobility remains a critical epidemiological vulnerability. Informal and cross-border transhumance networks facilitate the rapid dissemination of infected animals and vectors into unaffected regions, complicating surveillance and outbreak tracing [8]. Compounding this is the high genetic diversity of African CCHFV strains; frequent reassortment events and phylogenetic heterogeneity pose significant challenges for diagnostic accuracy, vaccine efficacy, and our understanding of viral virulence [12,122]. Genomic surveillance capacity, however, remains concentrated in a few regions, leaving significant gaps in our understanding of strain evolution [121]. Furthermore, the role of wildlife in maintaining CCHFV circulation is increasingly recognized but poorly characterized. The detection of viral RNA and antibodies in peridomestic rodents and other wildlife in Kenya and Mauritania suggests complex transmission cycles where certain species may act as bridge hosts or amplifiers between ticks and livestock [6,15].

#### 8.6.3. Preparedness and Research Gaps

Significant research and operational gaps hinder effective preparedness. The true disease burden is likely underestimated due to surveillance systems that fail to capture mild or remote infections [32]. Additionally, there is a lack of comparative implementation evidence for interventions such as tick control and community-based One Health models in African settings [90]. Vaccine development remains a major challenge, with no licensed vaccine currently available [7,73,88]. Although recent progress in interferon-deficient mouse models has expedited testing, development is constrained by viral genetic diversity and a lack of commercial interest beyond specific initiatives such as the WHO R&D Blueprint [15]. Even if a vaccine candidate is approved, manufacturing and deployment in high-risk African populations present substantial logistical and financial hurdles [101,123]. Addressing these multifaceted threats requires shifting from reactive outbreak response to proactive investments in zoonotic surveillance, laboratory infrastructure, and regional coordination [15]. Sustainable preparedness will require integrated One Health frameworks that can adapt to the shifting transmission dynamics of CCHF across diverse African ecosystems [30].

## 9. Prioritized Strategic Recommendations and Future Directions

### 9.1. Strengthening Integrated Surveillance and Diagnostic Systems

The evidence synthesized in this review indicates that passive surveillance and limited laboratory capacity contribute substantially to the under recognition of CCHFV transmission across Africa, particularly in remote pastoral and livestock-dependent regions [1,2]. Strengthening decentralized molecular and serological diagnostic capacity and integrating human, livestock, and vector surveillance through a One Health framework could improve early detection and outbreak response [18,31]. Community-based surveillance involving frontline health workers and livestock owners may enhance case detection in underserved settings, provided that appropriate training and coordination mechanisms are established [43]. Overall, the evidence supports strengthening integrated surveillance and laboratory networks within routine national public health and veterinary systems to improve long-term preparedness and reduce reliance on reactive outbreak responses [1].

### 9.2. Improving Outbreak Preparedness and Healthcare Response Capacity

The evidence reviewed indicates that delayed case recognition, limited diagnostic capacity, and weak surveillance systems remain major barriers to timely CCHFV outbreak detection and response across Africa [1,24]. Because the clinical presentation of CCHF frequently overlaps with other febrile illnesses, strengthening healthcare worker capacity in case recognition, specimen handling, and infection prevention and control (IPC) is essential to improve early diagnosis and reduce healthcare-associated transmission [94]. The review also highlights the need to strengthen decentralized response capacity and coordination between human and animal health sectors, particularly in remote pastoral settings where diagnostic access remains limited [1,24]. Sustained investment in laboratory infrastructure, IPC systems, healthcare workforce training, and peripheral health facilities should therefore form part of routine health system strengthening to improve outbreak preparedness and early case management across endemic regions [1,24,94].

### 9.3. Operationalizing One Health Preparedness Systems

While the evidence reviewed supports the importance of a One Health approach for CCHFV preparedness, its implementation remains uneven across many African settings [75,76]. The extensive movement of livestock and pastoralist populations across regions such as the Sahel and East African corridors underscores the need for stronger cross-border surveillance and coordination between human and animal health sectors [8,72,112]. Strengthening regional collaboration through existing platforms, including Africa CDC, could improve information sharing and coordination in outbreak response [8]. In addition, sustained One Health implementation will require institutional integration, workforce capacity building, and greater domestic investment to support long-term surveillance and preparedness beyond externally funded initiatives [26].

### 9.4. Strengthening Vector Control and Environmental Risk Reduction

The evidence synthesized in this review highlights the central role of *Hyalomma* ticks and livestock–human interfaces in CCHFV transmission across many African ecosystems, supporting vector control as a key component of prevention strategies [1,2,24]. Strengthening integrated tick control through veterinary services, appropriate acaricide use, and community engagement may reduce livestock infestations and human exposure in high-risk pastoral settings [124]. The review also demonstrates that ecological suitability alone does not necessarily reflect active viral transmission; therefore, environmental risk mapping should be integrated with human, livestock, and entomological surveillance to improve identification of areas with active circulation and guide targeted interventions [1,6]. Such integrated One Health approaches are likely to strengthen sustainable CCHFV preparedness across Africa [1,2,24].

### 9.5. Research, Innovation, and Vaccine Priorities

The evidence reviewed highlights substantial knowledge gaps in the epidemiology, transmission ecology, and surveillance of CCHFV across many African regions, underscoring the need for context-specific research to better define disease burden and transmission dynamics [2,5]. Priority areas include strengthening molecular surveillance, evaluating integrated One Health surveillance systems, and assessing the effectiveness of vector control and livestock-based sentinel approaches under routine field conditions [1,9]. Given the diagnostic challenges identified throughout this review, developing affordable and field-adapted diagnostic technologies suitable for decentralized settings remains an important research priority [92,101]. In addition, despite ongoing research into vaccine candidates, no licensed CCHFV vaccine is currently available, highlighting the need for continued investment in vaccine development and equitable access for populations at highest risk [9,17,122,123]. Overall, sustained investment in locally relevant research, surveillance, laboratory capacity, and local vaccine production will be essential to strengthen long-term CCHFV preparedness and response across Africa [122,123,124,125].

## 10. Conclusions

CCHFV poses a persistent and evolving One Health threat across Africa, driven by complex interactions among humans, livestock, vectors, wildlife, and environmental systems. Current evidence suggests that CCHFV circulation is far more widespread than reported human case data indicate, with many regions likely experiencing silent or underrecognized transmission. The continent’s diverse ecological and socioeconomic contexts might contribute to heterogeneity in hotspot distribution, outbreak dynamics, and preparedness capacity. Although important progress has been made in surveillance, molecular diagnostics, and outbreak response in several countries, major gaps remain in decentralized laboratory capacity, integrated surveillance, infection prevention and control systems, and sustainable preparedness infrastructure. Surveillance systems continue to operate largely in reactive modes, often identifying outbreaks only after severe human disease has emerged. Weak coordination among public health, veterinary, and environmental sectors further limits early detection and rapid containment.

The review underscores the urgent need to shift from fragmented, donor-dependent outbreak responses to institutionalized, multisectoral preparedness systems grounded in One Health principles. Strengthening regional laboratory networks, expanding livestock and entomological surveillance, improving rural healthcare readiness, enhancing community-based early-warning systems, and investing in integrated digital surveillance platforms are critical priorities for future preparedness. Future efforts must also prioritize operational research, genomic surveillance, validation of context-specific diagnostic tools, and the development of affordable point-of-care technologies suitable for resource-constrained settings. Given ongoing ecological changes linked to climate variability, land-use transformation, and transboundary livestock movement, proactive preparedness strategies will become increasingly important for mitigating future spillover risks.

Ultimately, effective CCHF control in Africa will depend not only on improved diagnostics and outbreak response but also on sustained political commitment, domestic investment, regional collaboration, and the long-term integration of human, animal, and environmental health systems.

## Data Availability

No new data were created or analyzed in this study. Data sharing is not applicable to this article.
